# Sinonasal Orbital Apex Syndrome, Horner Syndrome and Pterygopalatine Fossa Infection: A Case Report and Mini-Review

**DOI:** 10.3390/life13081658

**Published:** 2023-07-29

**Authors:** Gregorio Benites, Jure Urbančič, Carolina Bardales, Domen Vozel

**Affiliations:** 1 Departamento de Cirugía, Facultad de Medicina, Universidad Nacional de Trujillo, Roma Avenue 338, Trujillo 13001, Perumbardales@unitru.edu.pe (C.B.); 2 Faculty of Medicine, University of Ljubljana, Vrazov trg 2, 1000 Ljubljana, Slovenia; jure.urbancic@kclj.si; 3 Department of Otorhinolaryngology and Cervicofacial Surgery, University Medical Centre Ljubljana, Zaloška 2, 1000 Ljubljana, Slovenia; 4 Departamento de Cirugía, Especialidad de Otorrinolaringología, Hospital Belen de Trujillo, Bolivar Street 350, Trujillo 13001, Peru

**Keywords:** sphenoid sinusitis, skull base, ocular infections, sphenopalatine neuralgia, surgical endoscopy, osteomyelitis, case report

## Abstract

This paper presents a literature review and a case of an 83-year-old otherwise healthy female patient with a history of recent syncope, a sudden-onset right-sided temporal headache, diplopia, and vision loss. An exam revealed right-sided upper eyelid ptosis, myosis, vision loss, ophthalmoplegia, and a positive relative afferent pupillary defect on the right eye. CT showed sphenoid sinus opacification, eroded lateral sinus wall, Vidian canal, disease extension to the posterior ethmoid air cells, orbital apex, medial orbital wall, and pterygopalatine fossa. An orbital apex syndrome (Jacod’s syndrome), Horner syndrome, and pterygopalatine fossa infection were diagnosed due to the acute invasive fungal sinusitis developed from a sphenoid sinus fungal ball. The patient was treated with antimicrobial therapy and transnasal endoscopic surgery twice to decompress the orbital apex, drain the abscess and obtain specimens for analysis. The right-sided ptosis, visual loss, ophthalmoplegia, and headache resolved entirely. No immune or comorbid diseases were identified, microbiological and histopathological analyses were negative, and MRI could not be performed on the presented patient. For that reason, the diagnostic procedure was non-standard. Nevertheless, the treatment outcome of this vision and life-threatening disease was satisfactory. Treating the fungal ball in an older or immunocompromised patient is essential to prevent invasive fungal rhinosinusitis and fatal complications.

## 1. Introduction

Rhinosinusitis (RS) is an inflammatory disease of the nose and paranasal sinuses with a clinical picture of anterior or posterior nasal discharge, congestion, olfactory dysfunction, and facial pain or pressure. It must be confirmed with endoscopic or radiological signs of inflammation. By its duration, it is classified into acute (i.e., duration <4 weeks), subacute (4–12 weeks), and chronic RS (>12 weeks). Moreover, RS can be named according to the inflamed paranasal sinus, e.g., sphenoiditis in sphenoid sinus inflammation. RS significantly affects the patient’s quality of life due to the significant impact on personal development, sleep hygiene, mental health, physical condition, self-perception, and family relationships [1,2,3].

Acute RS commonly arises as a viral infection, which may present as a brief episode or prolonged disease due to an impaired mucociliary clearance caused by viruses. Impaired mucociliary clearance predisposes a mucosa to a bacterial or fungal superinfection [4,5]. Acute bacterial RS is most commonly caused by *Streptococcus pneumoniae*, *Haemophilus influenzae* type B, *Moraxella catarrhalis*, *Staphylococcus aureus*, and some anaerobic bacteria [6]. *Aspergillus* spp. are the most common causative agents of the fungal ball, a chronic RS, and the most common type of fungal RS—other fungi, e.g., *Mucor* spp. and *Rhizopus* spp. rarely cause fungal RS but typically present a type of acute fulminant invasive fungal RS called mucormycosis [7]. Less often, an invasive fungal RS can be caused by *Candida* spp., *Alternaria* spp., *Scadesporium* spp., and *Fusarium* spp. [8].

RS complications are rare, especially the ones derived from chronic RS. They can be divided according to the location into intracranial and extracranial (Table 1). The latter can be divided into orbital, orbital apex, bony and other complications. The infection of the skull base or regions outside the confines of paranasal sinuses (i.e., invasive form) and the involvement of the central nervous system are more common in immunocompromised than immunocompetent patients [9,10,11,12].

Since there is a paucity of literature describing rare and severe complications of RS, this paper presents a literature review (Table 2) and a case of a patient with an orbital apex syndrome (Jacod’s syndrome), Horner syndrome, and pterygopalatine fossa infection due to the acute invasive fungal RS, which developed from sphenoid sinus fungal ball. No immune or comorbid diseases were identified, microbiological and histopathological analyses were negative, and MRI could not be performed on the presented patient. The case report is presented per the CARE guidelines (Supplementary Materials).

## 2. Case Presentation

### 2.1. Initial Presentation and Treatment

**Figure 1:**
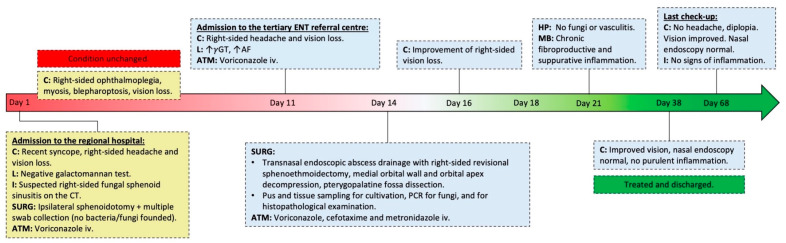
Case timeline of the patient with orbital apex syndrome, Horner syndrome, pterygopalatine fossa infection, and sphenoid sinusitis. C—clinical picture; L—laboratory test results; I—imaging studies results; SURG—surgical treatment; ATM—antimicrobial therapy; iv—intravenously; ↑—elevated levels; *γ*GT—gamma-glutamyl transferase; AF—alkaline phosphatase; PCR—a polymerase chain reaction.

An 83-year-old otherwise healthy female patient with a hip prosthesis was admitted to the regional secondary otorhinolaryngology service due to the history of a sudden-onset right-sided temporal headache followed by syncope, diplopia, and vision loss. Neurological and ophthalmological exams revealed right-sided upper eyelid ptosis, myosis, vision loss, ophthalmoplegia, and a positive relative afferent pupillary defect on the right eye (Figure 1).

**Figure 2:**
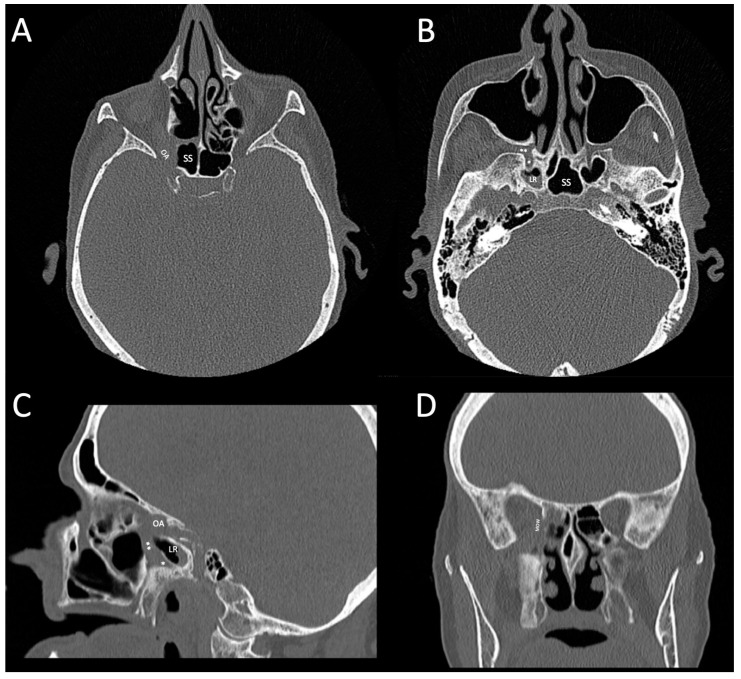
Initial CT prior to the first surgical intervention at the regional secondary otorhinolaryngology service showed sphenoid sinus opacification and the involvement of different adjacent structures. (**A**): axial CT shows the dehiscent lateral wall of the right sphenoid sinus (SS) and medial orbital apex (OA); (**B**): axial CT shows widened right Vidian canal (*), pterygopalatine fossa (**) and mucosal thickening in the lateral recess (LR) of the sphenoid sinus; (**C**): sagittal CT shows eroded superior part of right Vidian canal and mucosal thickening of the lateral recess of sphenoid sinus; (**D**): coronal CT shows mucosal thickening in the right posterior ethmoid sinus and dehiscence of medial orbital wall.

High-resolution CT of the head, skull-base, and paranasal sinuses suspecting stroke, performed in the regional emergency centre, showed right-sided high-density sphenoid sinus opacification with an eroded lateral sinus wall, Vidian canal, disease extension to the posterior ethmoid air cells, orbital apex, medial orbital wall, and pterygopalatine fossa (Figure 2). Due to the syncope, a CT angiography of the aortic arch, cervical and intracranial arteries was performed, which revealed no lesions in the central nervous system. MRI could not be performed due to the hip prosthesis. 

According to clinical and radiological findings, the diagnosis was a combined invasive fungal and bacterial sphenoid sinusitis complicated as an orbital apex syndrome, Horner syndrome, and pterygopalatine fossa infection. 

An otorhinolaryngologist was consulted, and the patient promptly underwent surgical treatment via transnasal endoscopic technique. A right-sided sphenoidotomy and posterior ethmoidectomy were performed. A fungal ball and pus were removed from the sphenoid sinus. Specimens were sent for microbiological analyses, which were non-diagnostic.

Immediately postoperatively, a loading dose of intravenous voriconazole was administered empirically and continued with 200 mg/12 h intravenously based on radiological, clinical, and intraoperative findings.

However, the patient’s clinical picture did not improve, and the patient was urgently transferred to the national tertiary referral otorhinolaryngology centre for further treatment ten days later. 

### 2.2. Revisional Treatment 

**Figure 3:**
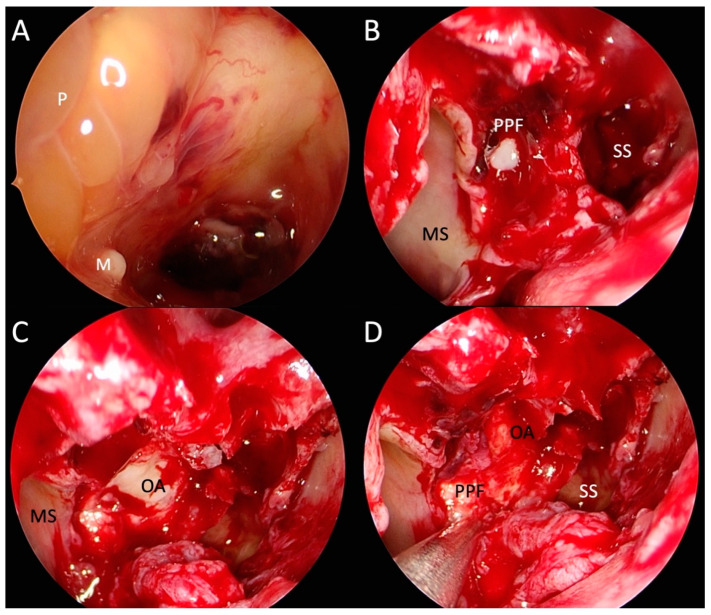
Photographs of transnasal endoscopic surgical treatment of invasive fungal rhinosinusitis causing right-sided orbital apex syndrome, Horner syndrome and pterygopalatine fossa infection. (**A**): right sphenoid sinus (SS) after sphenoidotomy, with polypoid (P) mucosa on its lateral wall obstructing lateral recess of sphenoid sinus and microabscess (M) on the sphenoid sinus floor; (**B**): view after right-sided extended middle meatal antrostomy showing maxillary sinus (MS), sphenoethmoidectomy and drainage of pus from pterygopalatine fossa (PPF); (**C**): decompression and drainage of the orbital apex (OA); (**D**): right pterygopalatine fossa and orbital apex after the decompression and drainage.

In the national tertiary otorhinolaryngology referral centre, an urgent revisional endoscopic right-sided sphenoethmoidectomy, middle meatal antrostomy, pterygopalatine fossa dissection, partial orbital decompression, and drainage of an inferomedial orbital apex were performed (14th day, see Figure 3). Swabs and tissues were collected for microbiological and histopathological analyses. 

Microbiological analysis from surgically obtained specimens identified no bacteria or fungi, and eubacterial PCR results were positive but non-diagnostic. Other blood analysis results were normal. A serum galactomannan value was within the normal range. CRP and erythrocyte sedimentation rates were slightly raised to 20 mg/L and 39 mm/h, respectively. The histopathological analysis identified no microbes or vasculitic changes but described the unspecific chronic fibroproductive and suppurative inflammation.

Immediately after the revisional surgery, cefotaxime 2 g/6 h i.v. and metronidazole 500 mg/12 h i.v. were added to the voriconazole 200 mg/12 h i.v. Due to the negative blood, microbiology and histopathology results, antimicrobial treatment was not changed until discharge from the hospital. An infectious disease specialist was consulted.

### 2.3. Follow-Up and Outcome

After the revisional surgery, the right-sided ptosis, visual loss, ophthalmoplegia and headache gradually improved by the 16th day; and resolved entirely until the discharge on the 38th day. Moreover, an endoscopy showed no signs of infection. A follow-up CT, performed on the 68th day, showed no residual inflammation. The patient was satisfied with the treatment.

## 3. Discussion and Literature Review

This paper presents a rare case of an orbital apex syndrome, Horner syndrome and pterygopalatine fossa infection as a consequence of acute invasive fungal sinusitis evolved from a fungal ball, which manifested with a syncope, unilateral visual loss, diplopia and temporal headache.

### 3.1. The Clinical Picture of Fungal Rhinosinusitis 

The sphenoid sinus is rarely involved in invasive fungal rhinosinusitis. Nonetheless, the acute, chronic, or acute fulminant form that can develop present a higher morbidity and mortality rate compared to other paranasal sinuses due to the proximity of vital neurovascular structures to the sphenoid sinus (internal carotid artery, cavernous sinus with contents, pituitary gland, optic nerve) [29]. 

Asymptomatic clinical picture and long-term indolent disease are more typical for non-invasive than invasive fungal RS. Among more or less troublesome symptoms could also be a bad smell in the saprophytic fungal infection, facial pain, post-nasal drip and cacosmia in the fungal ball, and proptosis in the allergic FR. There could be a slower progressive appearance of bloody nasal discharge, unilateral nasal obstruction, cacosmia, eye proptosis and sinonasal tumour-like lesion in the chronic invasive fungal RS. Conversely, the acute and subacute invasive fungal RS are clinically rich. They start as a short prodrome followed by rhinorrhea, nasal congestion, facial pain or pressure, and fever in the acute invasive fungal RS [30,31]. Fungal sphenoid sinusitis is usually asymptomatic in the early stages, delaying the diagnosis [19]. An unspecific or even florid clinical picture can support the possibility of fungal infection.

### 3.2. Orbital Complications of Sinonasal Disease

The spread of the infection to the orbit is uncommon in acute RS but occurs more frequently than the intracranial spread. Children and immunocompromised adults are more commonly affected. The predisposing factor is most commonly a dehiscence in the *lamina papyracea* in the posterior ethmoid, maxillary or frontal sinus due to an acquired bony erosion. A fungal orbital infection could also result from a venous spread [6,13,29]. The cases of orbital cellulitis and abscess occur mainly in the retrobulbar compartment of the orbit [32], e.g., in the orbital apex. 

Orbital apex refers to the most posterior part of the orbit. Numerous neurovascular structures communicate with it through the superior orbital fissure and the optic canal. Disorders of orbital apex comprise three syndromes depending on the lesion’s location: orbital apex syndrome, superior orbital fissure syndrome (Rochon-Duvigneaud syndrome) and cavernous sinus thrombosis (CST) (Table 1) [11]. Orbital apex syndrome encompasses CN II, III, IV, V_1_ and VI impairment due to inflammatory damage or direct compression [10]. Superior orbital fissure syndrome is caused by a lesion immediately anterior to the orbital apex. Its clinical presentation resembles the orbital apex syndrome. However, it lacks the CN II impairment. Cavernous sinus thrombosis presents with cheek and lower eyelid hypesthesia (both caused by damage to the CN V_2_) apart from the OAS signs. In addition, cavernous sinus thrombosis can be accompanied by Horner syndrome [32]. The latter evolves if the pericarotid sympathetic plexus is damaged anywhere along the course of the internal carotid artery, including its cavernous segment. It typically comprises ipsilateral upper eyelid ptosis, myosis and sometimes facial anhidrosis due to the damage in the sympathetic pathway. Pupillary responses are normal [12,16]. Another possible cause of diplopia is the myositis of extraocular muscles [32]. 

Our patient had eroded and dehiscent bony walls of the right sphenoid sinus, eroded and expanded Vidian canal and dehiscent posterior wall of the pterygopalatine fossa. The lateral recess of the sphenoid sinus was filled with swollen mucosa and mucus. The pterygopalatine fossa was inflamed, expanded and widely communicated with the orbital apex via an inferior orbital fissure and with the lateral recess of the sphenoid sinus via dehiscence through the posterior wall. Most observed changes can result from a fungal ball evolving into invasive fungal RS causing orbital apex syndrome, pterygopalatine fossa infection and Horner syndrome [24]. The pathophysiology of clinical features is described in Table 3. 

### 3.3. Risk Factors and Pathogenesis of Invasive Fungal Rhinosinusitis

There were no obvious identified risk factors for the development of invasive fungal RS as the patient did not have immunodeficiency or disease such as diabetes mellitus [25] or haematological malignancy [18]. These findings are consistent with the previously reported cases of invasive rhino-orbital-cerebral aspergillosis and invasive oronasal aspergillosis in immunocompetent patients [19,21,22,23]. 

According to the literature, a patient’s high age cannot be identified as the only risk factor contributing to the development of chronic RS. However, presbynasalis, which consists of collagen and nasal mucosa atrophy, a decrease in mucociliary transport, mucus production, the loss of vessel patency, and especially immunosenescence in older patients are considered a significant determinant in the development of RS [8,34].

Our patient had no classic environmental or occupational fungi exposure (e.g., working or living in a moist environment, exposure to construction or excavation sites) according to the epidemiological enquiry performed by the infectious disease specialist. However, the patient’s residence was not examined, which could reveal the link between domestic mould exposure and invasive aspergillosis as already described elsewhere [35]. Nevertheless, it has been stated that fungi are almost ubiquitous in the paranasal sinuses. After inhaling the spores, their pathogenicity depends more on the patient’s state than the fungi [30,36]. If the fungi had passed the epithelium, the infection could be considered invasive and vice versa. Therefore, even extensive growth of the fungi could be only saprophytic colonization or fungal ball on one side of the spectrum or a fatal invasion of the central nervous system in invasive fungal RS on the other side of the spectrum [29]. Namely, the fungal ball can progress to acute invasive fungal RS [23] or to micro-invasive form, which was recently termed intermediate invasive fungal RS [24]. This is a possible explanation of pathogenesis in our patient. The hallmark of acute-onset severe immune depression is diabetic ketoacidosis, which poses a threat to developing a mucormycosis caused typically by *Mucor* spp. and *Rhizopus* spp. [6,31]. 

### 3.4. Microbiology and Histopathology Analyses

Microbiological analyses from the transnasally collected specimen are considered in diagnosing acute RS when the treatment fails [6,14,37,38]. The efficacy of pus specimen cultivation in acute RS varies between 33% and 66% [6,14,37]. Moreover, the rate of positive results for fungal cultivation is only 33% due to microinvasion of vascular tissues, which results in negative results [8,19,37]. Histopathological analyses can be negative in the presence of sparse fungal forms, which is typical in chronic invasive fungal RS [28]. In addition, microbiological and histopathological analyses yield depends upon properly sampling the infected tissue. Therefore, negative fungal cultures and histopathology results do not rule out the presence of fungal infection [28,31]. When the infection invades the skull base and presents as atypical skull-base osteomyelitis, obtaining the specimen to perform analyses may be even more challenging. It is paramount to put a multidisciplinary team effort into managing these cases [9,13,17].

In the presented case, a microbe was not identified microbiologically or histopathologically after two surgeries. Moreover, systemic markers of fungal infection (galactomannan, beta-D-glucan) were negative. Nevertheless, the fungus was observed macroscopically intraoperatively as a fungal ball in the sphenoid sinus. Due to the clinical and radiological characteristics of orbital apex syndrome, Horner syndrome, and pterygopalatine fossa infection, the patient’s diagnosis was an acute invasive fungal sinusitis treated empirically. PCR of tissue samples obtained during the first surgery at the regional secondary otorhinolaryngology service could confirm the presence of fungi as already described elsewhere [17,28] but were unfortunately not performed. The lack of PCR is the main limitation of our report and patient management. It is possible that microbiological and histopathological analyses obtained during the second surgery were negative due to the previous initiation of antimicrobial therapy and other already described reasons related to microbiology and histopathology analyses [6,8,14,19,37]. Moreover, tissue sampling was more challenging than the first surgery, when the fungal ball was observed macroscopically. Since the patient’s condition improved after the second surgery, no debridement and second sampling were performed, as in other studies [28]. 

### 3.5. Surgical Treatment and Postoperative Management

Complications of RS need to be treated aggressively, either conservatively and/or surgically. The decision for surgical treatment depends on the clinical picture and radiological findings. Nevertheless, appropriate broad-spectrum intravenous antibiotics must be initiated to cover staphylococci, streptococci, and anaerobes. Eventually, culture and laboratory test results should guide the definite therapy [13].

Surgical intervention can be performed via a transnasal endoscopic approach for medially located lesions, e.g., medial to the sagittal mid-pupillary line. A transorbital approach (e.g., via blepharoplasty or lateral decompression incision) should be utilized for laterally located lesions. Other transorbital approaches for the medially located disease include the Lynch–Howarth or transcaruncular incision, the transconjunctival approach, and a combined external and transnasal endoscopic approach. The endoscopic technique can be used transorbital as well [13]. Nowadays, in a sinonasal orbital or orbital apex complication, the transnasal endoscopic approach presents a standard treatment since it enables the causal treatment of orbital disease (i.e., paranasal sinus drainage in endoscopic sinus surgery) [26]. 

A transnasal endoscopic unilateral sphenoethmoidectomy, pterygopalatine fossa dissection, medial orbital wall and orbital apex decompression were performed efficiently in our case. The indication for the surgery was clear since there were no symptoms resolution days after the first surgery, and the different potential pathologies caused the patient’s clinical picture. In the case of acute invasive fungal RS, an approach considering surgery and antifungal antibiotics is recommended to make a debridement and take additional samples, which was utilized in our patient. Using a correct early antifungal therapy improves the outcome in these patients. Hyperbaric oxygen therapy is also an option. However, it was deemed unnecessary in our case [31].

## 4. Conclusions

Orbital apex syndrome is a rare disease that should be suspected in every patient with a history of sinonasal disease, visual deterioration, diplopia, unilateral headache or facial pain. Horner syndrome can develop if there is a spread to the pericarotid sympathetic plexus. It is essential to recognize and treat the disease promptly to prevent permanent vision loss, diplopia and fatal complications, including death, especially in cases with limited radiological availability and negative microbiological and histopathological results. Among various etiological factors of orbital apex syndrome, invasive fungal rhinosinusitis should not be overlooked, especially in immunocompromised and elderly patients. In these patients, treating the fungal ball of the paranasal sinus early to prevent its progression to invasive fungal rhinosinusitis is essential.

## Figures and Tables

**Table 1:** Classification of rhinosinusitis complications [6,9,10,11,12,13,14,15,16,17].

Extracranial				Intracranial (~17.5%)
Orbital (~70%)	Bony (~5%)	Orbital Apex (~3.7%)	Other (~3.7%)	
Preseptal cellulitis	Pott’s puffy tumour	Orbital apex syndrome (Jacod’s syndrome)	Mucopyococele	Epidural abscess
Postseptal cellulitis	Atypical skull-base osteomyelitis	Superior orbital fissure syndrome	Pterygopalatine fossa infection	Subdural empyema
Subperiosteal abscess		Horner syndrome	Infratemporal fossa infection	Meningitis
Orbital abscess			Sepsis	Intraparenchymal abscess
Cavernous sinus thrombosis			Deep neck infection	Willis circle artery mycotic aneurism

Cavernous sinus thrombosis usually comprises CN III, IV, V_1_, V_2_, VI dysfunction and Horner syndrome x. Orbital apex syndrome comprises CN II, III, IV, V_1_, and VI dysfunctions. Superior orbital fissure syndrome comprises CN III, IV, V_1_, and VI dysfunctions. Mucopyocele typically occurs in the frontal sinus. CN—cranial nerve.

**Table 2:** Cases of rhinosinusitis complications reported in the literature.

Reference	Nr.	Sinus	Complications	Identified Microbe
Carta et al. (1998) [18]	1	Sphenoid	Cavernous sinus thrombosis	*Aspergillus fumigatus*
Lee et al. (2014) [19]	12	Sphenoid	Orbital apex syndrome, cavernous sinus thrombosis, parapharyngeal, pterygopalatine fossa, masticator space, intracranial infection	*Aspergillus* spp., *Mucor* spp.
See et al. (2016) [20]	1	Sphenoid	Cavernous sinus thrombosis, orbital apex, dura, Meckel’s cave and clivus infection	Unidentified
Warburton et al. (2016) [13]	1	Maxillary	Orbital apex syndrome, orbital compartment syndrome, orbital cellulitis	Unidentified
Käcker et al. (2019) [12]	1	Sphenoid	Horner syndrome, cavernous sinus, ophthalmic vein, superior petrosal sinus, sigmoid sinus, internal jugular vein, and sphenoparietal sinus thrombosis	*Staphylococcus aureus*
Lee et al. (2021) [15]	1	Pansinusitis	Stroke, sepsis, intracranial, bilateral retropharyngeal and bilateral longus colli muscle abscess, bilateral cavernous sinus and left ophthalmic vein thrombosis	*Staphylococcus hominis*
Costa Paiva et al. (2020) [21]	1	Oral cavity	Invasive fungal nasal cavity and hard palate infection	*Aspergillus* spp.
Leroy et al. (2020) [22]	1	Sphenoid, ethmoid	Orbital myositis, orbital apex infection, cavernous sinus thrombosis, cerebral abscess	*Aspergillus fumigatus*
Rissardo et al. (2020) [16]	1	Rhinosinusitis (sinus not specified)	Horner syndrome	Not reported
Assiri et al. (2021) [23]	10	Maxillary, sphenoid, ethmoid	Invasive fungal sinusitis, fungal ball, pterygopalatine and infratemporal fossa invasion, orbital and clivus invasion	*Aspergillus fumigatus*, *Staphylococcus epidermidis*, *Staphylococcus lugdunensis*, *methicillin-resistant Staphylococcus aureus*, *Klebsiella pneumonia*
Burnham et al. (2021) [24]	8	Sphenoid, ethmoid, maxillary, frontal	Invasive fungal sinusitis, invasion to orbital apex, optic nerve, pterygopalatine fossa, and clivus; intracranial and skull base invasion	*Aspergillus fumigatus*, *Rhizopus* sp. and *Scedosporium apiospermum*
Cullen et al. (2021) [25]	1	Sphenoid, ethmoid, maxillary	Orbital apex syndrome	*Staphylococcus aureus*, *Aspergillus fumigatus*
Yuan et al. (2021) [26]	2	Sphenoid	Cavernous sinus thrombosis, orbital apex syndrome, intracranial abscess, stroke	*Aspergillus fumigatus*
Lee et al. (2022) [27]	3	Unspecified	Orbital apex syndrome	Not reported
Ning et al. (2023) [28]	1	Maxillary, ethmoid	Invasive fungal sinusitis, orbital oedema	*Aspergillis fumigatus*

Nr.—number of cases.

**Table 3:** Main clinical features and pathophysiological correlations in our patient with orbital apex syndrome, Horner syndrome and pterygopalatine fossa infection [10,11,12,13,16,32,33].

Clinical Features	Pathophysiology
Vision loss, positive RAPD ^1^	Damage to the CN II at the optic disc or along the nerve trunk due to compression, ischemia or neuritis
Painful eye movement ^1^	Release of pro-inflammatory substances and/or rapidly increasing intra-orbital pressure
Periorbital pain ^1^	Inflammation of orbital contents
Ophthalmoplegia, diplopia ^1^	Loss of oculomotor CNs (III, IV, VI) innervation or impairment of extraocular muscles
Proptosis ^1^	Loss of globe’s extraocular muscle tension and/or retrobulbar swelling and/or venous congestion
Ptosis ^1,2^	Loss of CN III innervation to the *levator palpebrae superioris* muscle and/or loss of sympathetic innervation to the superior tarsal muscle
Mydriasis ^1^	Loss of CN III innervation to the sphincter dilator muscle
Ipsilateral forehead hypoesthesia and/or pain ^1^	Damage to the CN V_1_
Syncope	Oculocardiac reflex pressure due to the pressure on the optic nerve and/or tension of extraocular muscles
Loss of corneal reflex ^1^	Damage of the nasociliary nerve (branch of CN V_1_)
Myosis ^2^	Loss of sympathetic innervation to the pupil dilator muscle
Anhidrosis ^2^	Impaired sympathetic innervation of the facial sweat glands

^1^ Signs and symptoms that constitute orbital apex syndrome. ^2^ Signs and symptoms that constitute Horner syndrome. Signs of grey cells were not identified in our patient. Orbital apex syndrome can be caused by other than infectious aetiology (e.g., traumatic, neoplastic, inflammatory, vascular) [27]. RAPD—relative afferent pupillary defect; CN—cranial nerve.

## Data Availability

Not applicable.

## Supplementary Materials

The following are available online at https://www.mdpi.com/article/10.3390/life13081658/s1, Figure S1: CARE checklist.
